# Monitoring Acute Posterior Multifocal Placoid Pigment Epitheliopathy Disease Progression Using Non-invasive Multimodal Imaging: A Case Series

**DOI:** 10.7759/cureus.98460

**Published:** 2025-12-04

**Authors:** Andrew Palmier, Alastair Bezzina

**Affiliations:** 1 Ophthalmology, Mater Dei Hospital, Msida, MLT

**Keywords:** apmppe, case series, fundal autofluorescence, imaging, optical coherence angiography, optical coherence tomography, white dot syndromes

## Abstract

In this case series, we present two cases of acute posterior multifocal placoid pigment epitheliopathy (APMPPE): a patient presenting for the first time with headaches and prodromal symptomatology associated with mild photophobia who was later diagnosed with APMPPE, and a patient who presented with a relapse of the same disease entity who was recently started on mycophenolate mofetil in view of previous macular involvement. In both cases, disease progression and therapeutic effect were assessed using non-invasive imaging, including optical coherence tomography (OCT) and fundus photography. Imaging biomarkers in keeping with disease resolution included a reduction in hyper-reflectivity in the outer plexiform layer (OPL) and outer nuclear layer (ONL) overlying the active placoid lesions on OCT, a progression from heterogeneous autofluorescent foci to smaller hypo-autofluorescent ones demonstrating a reduction in the outer retinal infiltrate and emergent retinal pigment epithelium dysfunction as well as a reduction in choriocapillaris flow voids on OCT angiography as the clinical picture improved. This case series and accompanying literature review help demonstrate the use of non-invasive imaging modalities in monitoring disease progression and screening for new disease activity in APMPPE without requiring the use of frequent dye-based angiography studies.

## Introduction

Acute posterior multifocal placoid pigment epitheliopathy (APMPPE) is an uncommon inflammatory capillaropathy initially described by Gass et al. in 1968 (forming part of the “White Dot Syndrome” spectrum of diseases) [1]. APMPPE is classically bilateral and is more common in Caucasians with a slight female preponderance [2]. The pathophysiology of APMPPE is still being debated, but a vasculitic process primarily affecting the choriocapillaris layer is thought to be the main cause of the disease. This inflammation at the level of the choriocapillaris causes hypoperfusion with secondary ischemia of the overlying retinal pigment epithelium (RPE) and photoreceptor layer [3]. APMPPE is characterized clinically by a viral prodrome followed by the emergence of visual symptomatology, namely photopsia, scotomata, and, if the fovea is involved, metamorphopsia and a reduction in visual acuity. The disease is characterized by yellow placoid lesions involving the posterior pole and mid-periphery and may be associated with a mild vitritis [2]. APMPPE episodes are usually self-resolving, but treatment may be employed, namely the use of corticosteroids in the acute phase and the introduction of steroid-sparing agents in persistent cases and/or when the macula is at risk [4]. The aim of this report is to demonstrate the efficacy of non-invasive imaging, namely: optical coherence tomography (OCT), fundal autofluorescence (FAF) photography, and OCT angiography, in tracking disease progression in two cases of APMPPE without the need of employing dye-based angiography.

Although fluorescein angiography (FFA) and indocyanine green angiography (ICGA) remain important diagnostic tools, their repeated use is limited by invasiveness, risk of adverse reactions, and impracticality for close-interval monitoring. This creates a clear clinical need for reliable non-invasive imaging biomarkers that allow safe, frequent assessment of disease activity.

This case series addresses this gap by illustrating the longitudinal evolution of APMPPE using serial multimodal, non-invasive imaging. Both patients have provided their consent for the drafting and publication of this case series.

## Case presentation

Case report 1

**Figure 1:**
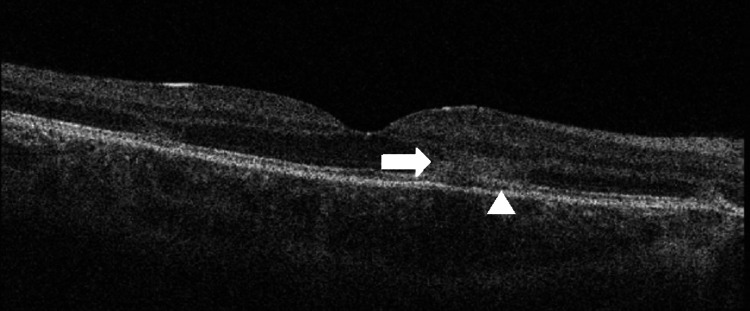
Optical coherence tomography (OCT) B-scan (foveal cut) demonstrating an inflammatory infiltrate in the peri-foveal area within the outer plexiform layer (OPL)/outer nuclear layer (ONL) area (arrow) with associated disruption down to the retinal pigment epithelium (RPE) layer (arrowhead).

**Figure 2:**
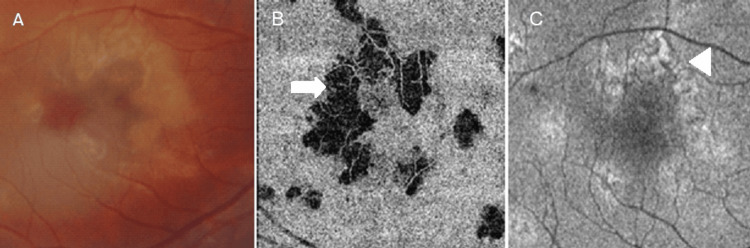
Yellowish placoid lesions noted in the perifoveal area of the right eye on fundoscopy (A). Optical coherence tomography (OCT) angiography demonstrated flow void areas at the level of the choriocapillaris (B, arrow) corresponding to the placoid area (A). Variable autofluorescence on fundal auto-fluorescence (FAF) (C), demonstrating early reduction in outer retinal infiltrate and unmasking of a hypo-autofluorescent retinal pigment epithelium (RPE) (arrowhead).

A 22-year-old gentleman, previously diagnosed with APMPPE in late 2021 and later started on mycophenolate mofetil in view of evidence of bilateral involvement and macular involvement in the left eye, presented to the eye casualty clinic in mid-October 2022 with a new onset of fever, headache, and lethargy, but did not report any new visual symptomatology. The patient was reviewed and was discharged as there was no evidence of active lesions in either eye. The patient returned after a few weeks, complaining of new-onset para-central scotomata in the right eye. Clinical examination revealed a best-corrected visual acuity of 6/9 Snellen in the right eye (reduced from 6/6 at his previous visit) and 6/36 in the left eye secondary to prior macular involvement. There was no evidence of anisocoria or an afferent pupillary defect. Ocular motility and confrontational visual fields were normal. Fundoscopy revealed small placoid lesions superior and nasal to the fovea in the right eye and mid-peripheral scars in both eyes. OCT analysis of the lesions revealed cells in the posterior vitreous and hyper-reflectivity in the outer plexiform layer (OPL) and outer nuclear layer (ONL) with disruption of the ellipsoid line and photoreceptor outer segment tips (Figure 1). Flow-voids in the choriocapillaris layer, corresponding to the placoid lesions noted on fundoscopy and variable autofluorescent areas on FAF, were noted on OCT-A (Figure 2).

**Figure 3:**
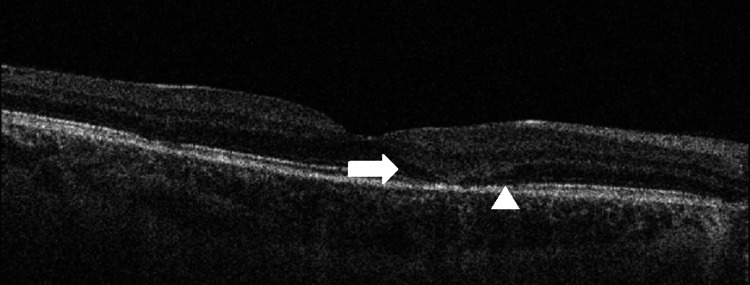
Follow-up optical coherence tomography (OCT) B-scan (foveal cut) of the right eye showing an attenuating infiltrate (arrow) in the outer plexiform layer (OPL)/outer nuclear layer (ONL) with a reappearance of the outer limiting membrane and inner segment-outer segment junction (IS-OS junction) (arrowhead).

**Figure 4:**
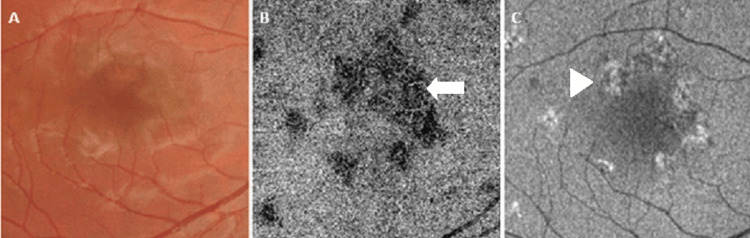
Follow multimodal imaging of the right macula. Attenuated placoid lesions with early hyperpigmentation sparing the fovea on fundoscopy (A). Corresponding choriocapillaris flow-void areas on OCT-A (B) have diminished on follow-up scanning with reconstitution of flow in the ischemic areas (arrow). A reduction in the size of altered autofluorescent areas consistent with a further reduction in outer retinal infiltrate and recovery of affected RPE (C, arrowhead).

The patient was started on systemic corticosteroid therapy, namely, prednisolone at 1 mg/kg/day, and was seen frequently in view of the risk posed to the right macula. During the subsequent visits, the patient was examined using bio-microscopy and by assessing the lesions using multi-modal imaging. Steady resolution of the lesions was noted starting from the first week after initiating corticosteroid therapy, with early changes noted on FAF in terms of increasing hyper-autofluorescence, suggestive of a reduction in the outer retinal layer infiltrate, allowing for hyperfluorescence of the diseased RPE layer to be transmitted. Subsequent visits revealed gradual attenuation of the hyperreflective infiltrate in the outer retinal layers overlying the placoid lesions (Figure 3) with increasing hyperautofluorescence and a reduction in the flow-void areas on OCT-A (Figure 4).

A viral screen and magnetic resonance imaging of the brain were performed to exclude central nervous system involvement and screen for contraindications to TNF-inhibitor treatment, which was later commenced in order to reduce the risk of relapse when considering the poor visual acuity in the left eye and the macula being at risk in the right. The patient’s visual acuity improved back to 6/6 Snellen in the right eye and remained stable in his left. A structured summary of the clinical course and corresponding multimodal imaging changes is presented in Table 1.

**Table 1:** Case 1 - Timeline of disease evolution on multimodal non-invasive imaging.

Timepoint	Findings
Week 1	Noticeable reduction in OPL/ONL hyperreflectivity; early restoration of the ellipsoid zone (EZ); FAF shows increased hyper-AF indicating reduced outer retinal infiltrate; OCT-A shows partial reduction in flow voids.
Weeks 2-3	Continued structural improvement; OPL/ONL infiltrate further attenuates; EZ becomes more continuous; flow restoration in choriocapillaris more evident; fundus lesions begin to pigment.
Month 1	Significant resolution of hyperreflective outer retinal material; marked narrowing of flow-void areas on OCT; FAF transitions from mixed AF to smaller discrete hypo-AF spots suggestive of early RPE dysfunction.
Final Outcome	Macula stabilized; no further lesion formation; imaging consistent with recovering outer retina and partial RPE remodeling.

Case report 2

**Figure 5:**
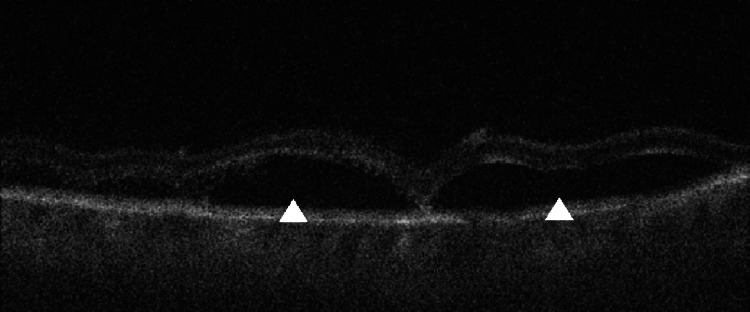
Optical coherence tomography (OCT) B-scan demonstrating a bacillary detachment (arrowheads) overlying one of the peripheral placoid lesions in the right eye.

A 31-year-old gentleman was admitted to the hospital in view of a one-week history of headaches and lethargy associated with blurred vision and mild photophobia. The patient did not have any past medical or ophthalmic history of note. The patient was initially investigated by the medical team, who diagnosed him with pre-renal failure secondary to dehydration based on his initial serological results. An ophthalmic review was requested in view of the blurring of vision, which persisted after admission. The initial examination revealed a best corrected visual acuity of 6/6 Snellen in both eyes, and there was no evidence of anisocoria and/or an afferent pupillary defect. Ocular motility and visual fields (by confrontation) were normal, and the patient managed to read all the Ishihara plates shown, suggesting an unremarkable neuro-ophthalmic status. Slit-lamp examination, however, revealed 1+ cells (SUN grading) with minimal-to-no flare in both eyes with no evidence of posterior synechiae or other iris abnormalities. Posterior segment examination revealed 1+ vitreous cells but no condensations or veils. Multiple hypopigmented plaque-like lesions were noted across the fundus in both eyes, with lesions noted in the vicinity of the macula bilaterally, more so in the left eye compared to the right. Bacillary retinal detachments were noted in the periphery (Figure 5).

**Figure 6:**
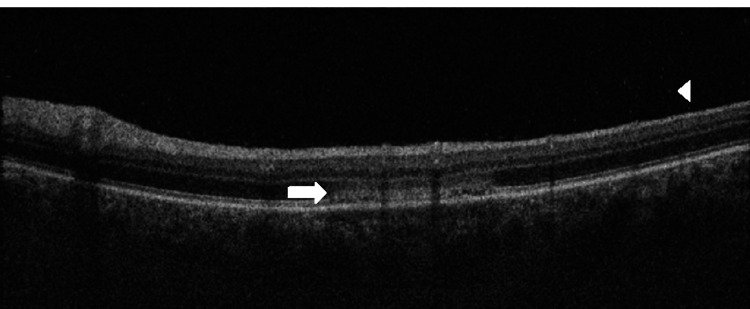
Optical coherence tomography (OCT) B-scan of the placoid lesion below the fovea of the left macula demonstrating an inflammatory infiltrate within the outer plexiform layer (OPL)/outer nuclear layer (ONL) region (arrow) as well as posterior vitreous cells (arrowhead).

**Figure 7:**
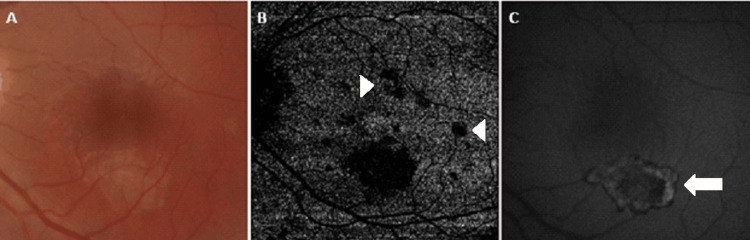
Multimodal images of the left macula acquired on presentation. Hypopigmented placoid lesions noted in the left eye (A) with corresponding flow void foci on OCT-A (B). Of note, one can appreciate multiple satellite lesions (arrowheads) around the fovea, not evident on fundoscopy or fundal autofluorescence (FAF). Hyper-autofluorescence around the border of the inferior lesion (arrowhead) noted on FAF, indicating early resolution (C).

OCT examination revealed increased hyperreflectivity in the OPL/ONL interface with an associated flow-void (Figure 6), noted on OCT-A, in the underlying choriocapillaris. FAF analysis showed a mixed hyper- and hypo-autofluorescent trace over the same lesions corresponding to the same plaques noted on fundoscopy (Figure 7). Further serological tests were performed to rule out other causes, including an interferon-gamma release assay for tuberculosis, serum lysozyme and angiotensin converting enzyme assays, syphilitic screen, human immunodeficiency virus assay, inflammatory markers, and high-resolution thoracic CT-scan, all of which came out negative. Based on fundal and OCT imaging and autofluorescence qualities, paucity of vitreous and anterior chamber inflammation, and exclusion of other etiologies associated with placoid chorioretinitis, the diagnosis of APMPPE was made, and a decision was taken to observe the patient, seeing that the patient was asymptomatic with evidence of resolution of the placoid lesions in the periphery and adjacent to the vascular arcades.

**Figure 8:**
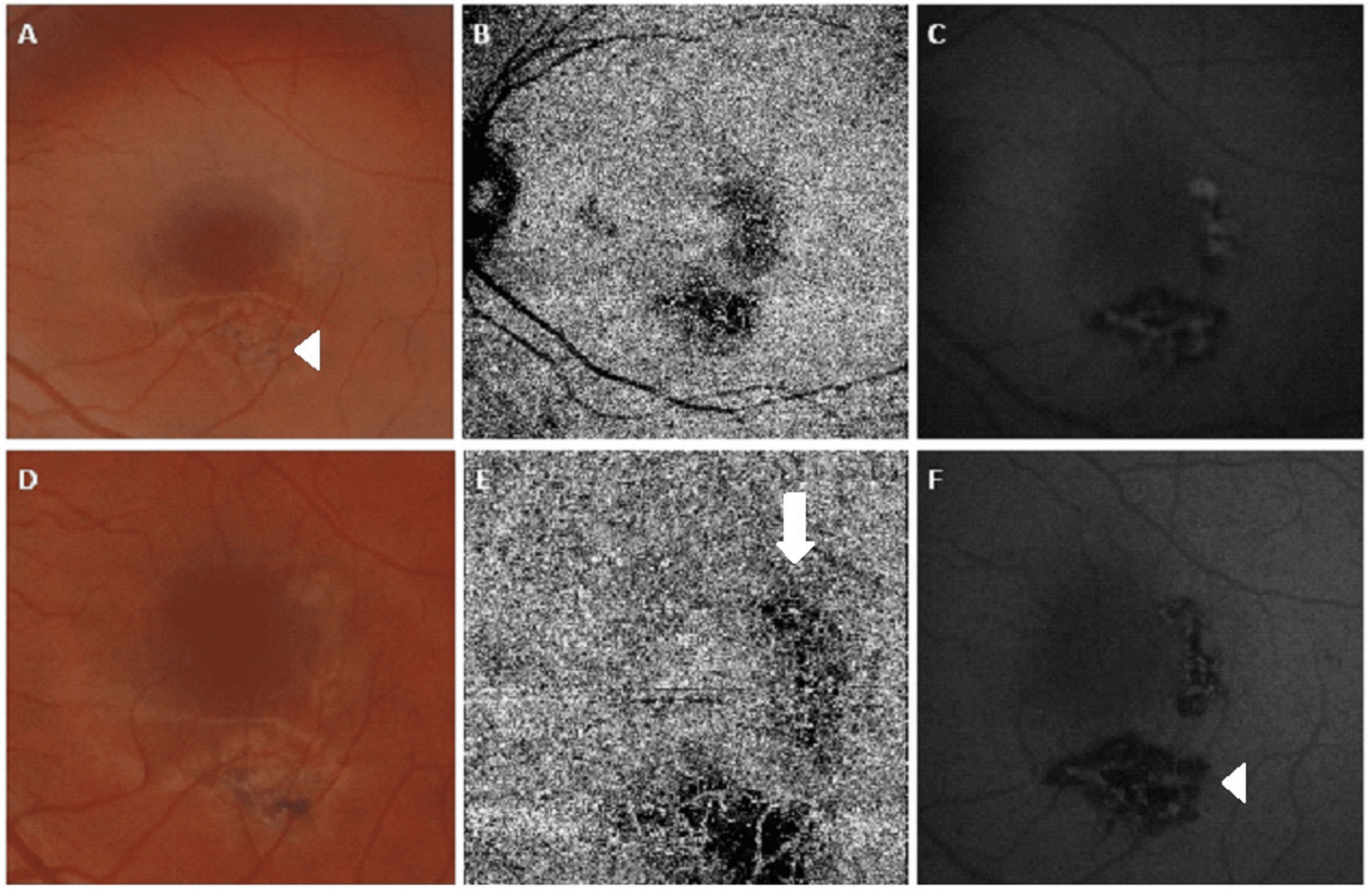
(A-C) The status of the left macular area one month following presentation. Increasing pigmentation over the inferior lesion (arrowhead) compared to the temporal lesion, which was still emerging one month prior. Increasing choriocapillaris vessel density can be observed (B) with mixed autofluorescence noted on FAF (C). (D-F) Further resolution of the placoid lesions around the left macula two months following presentation with increasing pigmentation in both lesions (D). Increased vessel density can be seen underlying the temporal perifoveal lesion (E, arrow) compared to one month before. (F) Increased hypo-autofluorescence in both lesions, suggesting a reduction in the inflammatory infiltrate within the outer layers of the neurosensory retina and atrophy of the retinal pigment epithelium (arrowhead).

After one month, there was a gradual decrease in outer retinal infiltrate on OCT with an associated reduction in choriocapillaris flow-void areas and increased vessel density on OCT-A, suggesting reperfusion. This was associated with a gradual increase in hyper-autofluorescence, with the area of the placoid lesion associated with a reduction in OPL/ONL hyper-reflectivity (Figure 8). After two months, the lesions had reached maximal attenuation on OCT-A and FAF with increased hypo-autofluorescence in the latter, suggesting retinal pigment epithelial loss (Figure 8). The patient has remained asymptomatic since diagnosis, with no evidence of new lesions on examination. A detailed timeline of imaging findings for Case 2 is shown in Table 2, followed by a comparative overview of both cases in Table 3.

**Table 2:** Case 2 - Timeline of disease evolution on multimodal non-invasive imaging.

Timepoint	Findings
Week 1	OPL/ONL hyperreflectivity begins to fade; OCT-A shows improving perfusion, though flow voids persist; FAF shows early hyper-AF rims around lesions, indicating outer retinal clearance.
Week 2	Satellite OCT-A lesions begin to shrink; vessel density improves around perifoveal areas; reduced inflammatory infiltrate on OCT; fundus lesions stabilize.
Month 1	Marked reduction in choriocapillaris flow-voids; peripheral lesions flatten and pigment; FAF pattern shifts to smaller hypo-AF areas; OCT shows near-complete normalization of OPL/ONL reflectivity.
Month 2	Maximal attenuation of lesions; OCT-A shows near-complete reperfusion except for focal residual voids; FAF shows stable hypo-AF patches consistent with limited RPE atrophy.
Final Outcome	Full clinical resolution; no recurrence; minimal non-progressive RPE changes.

**Table 3:** Comparative summary of baseline clinical and imaging features in two acute posterior multifocal placoid pigment epitheliopathy (APMPPE) cases.

	Case 1	Case 2
Demographics	22-year-old male	31-year-old male
History	Previous APMPPE with left macular involvement; on mycophenolate mofetil	No medical/ocular history
Systemic Symptoms	Fever, headache, lethargy	Headache, lethargy, mild photophobia
Presenting Ocular Symptoms	New paracentral scotomata (OD)	Blurred vision with preserved VA
VA at Presentation	OD: 6/6 ; OS: 6/36 (reduced from prior episode)	6/6 OU
Anterior/Vitreous Findings	No AC reaction; posterior vitreous cells (seen on OCT)	1+ AC cells; 1+ vitreous cells
Fundus Findings	Small placoid lesions nasal/superior to fovea	Multiple placoid lesions OU; peripheral bacillary detachment
OCT (Baseline)	OPL/ONL hyperreflectivity; EZ disruption	OPL/ONL hyperreflectivity; bacillary detachment
OCT-A (Baseline)	Choriocapillaris flow voids	Choriocapillaris flow voids + satellite lesions
FAF (Baseline)	Mixed hyper-/hypo-autofluorescence	Mixed autofluorescence; hyper-AF borders
Management	Prednisolone 1 mg/kg/day → TNF-inhibitor	Observation alone

## Discussion

APMPPE is a rare type of posterior uveitis that generally affects young adults who commonly present with rapid-onset blurred vision associated with central and paracentral scotomas and typically features bilateral creamy yellow placoid lesions located posterior to the equator. The exact sequence of events in the etiopathogenesis of APMPPE is still debatable. However, since Gass first described the condition in 1968 [1], the recent application of multimodal imaging has shifted the focus of its pathophysiology from the RPE to the choriocapillaris. Multimodal imaging features in APMPPE indicate a primary inflammatory involvement of the choriocapillaris with resultant photoreceptor disruption [2].

As can be demonstrated, in the two presented cases, non-invasive multimodal imaging acts not only as an ancillary test to confirm the diagnosis of APMPPE but also to monitor disease progression and the response to any therapy provided. Both cases in this series demonstrate imaging patterns that align closely with previously published descriptions of APMPPE evolution. Case 1 showed steroid-responsive reduction of OPL/ONL hyperreflectivity and progressive choriocapillaris reperfusion on OCT-A. Case 2 illustrated the hyperacute spectrum with bacillary detachment, an increasingly recognized manifestation, followed by spontaneous recovery. These findings reinforce the temporal relationship between choriocapillaris ischemia and secondary outer retinal injury.

Our findings on serial imaging further support the hypothesis that choriocapillaris hypoperfusion precedes outer retinal disruption, as the areas of OCT-A flow deficit consistently extended beyond zones of structural OCT abnormality early in the disease course.

On OCT, during the acute stage of the condition, the typical yellow-white placoid lesions appear as hyperreflective material at the outer retinal and RPE layers with disruption of the outer retinal and ellipsoid zone [5]. As the placoid lesions resolve, the outer retinal hyper-reflectivity disappears, resulting in focal areas of photoreceptor layer and RPE disruption or incomplete restoration of its appearance [6]. In 2012, Goldenberg outlined four distinct OCT phases of APMPPE. The initial phase is characterized by a prominent elevation corresponding to a placoid lesion resulting in a disruption of the photoreceptor outer segment tip layer and the presence of a variable amount of subretinal fluid. This is followed by a rapid flattening of the elevation and thickening of the inner-outer segment (IS/OS) junction and the ONL layer. The second stage occurs around two weeks later and is distinguished by the detection of a distinct separation between the IS/OS junction and the RPE. The third stage occurs around six weeks after disease onset and has the longest duration. It is characterized by accentuated RPE hyperreflectivity and an attenuation of the outer layers ranging from the IS/OS junction to the RPE layer. The fourth phase, also known as the resolution phase, occurs at three months post-disease onset, with the reappearance of two separate, distinguishable layers of photoreceptors and RPE [7]. Even though the photoreceptor and RPE layers almost regain their normal appearance, focal areas of atrophy can develop. In some cases of APMPPE, another well-described finding on OCT is the accumulation of subretinal fluid, and in rare cases, exudative retinal detachment. Kohli et al presented three cases of APMPPE with bacillary layer detachment in the acute phase. As observed in the second case, these OCT findings were short-lived and resolved completely within a week [8].

OCT-A is a valuable non-invasive imaging modality for this condition, and it has provided valuable insight into the role of impaired choroidal perfusion in the pathogenesis of APMPPE. OCT-A offers obvious advantages over other fluorescein and ICGA; it is non-invasive since it does not require intravenous dye injection and provides detailed images of the retinal vasculature in three dimensions. Furthermore, their repeated use in the monitoring of disease activity is time-consuming and costly, making OCT-A a more feasible and cost-effective option. Another major advantage of OCT-A compared to dye-based investigations is the lack of leakage, which is, in fact, also considered one of its greatest limitations. Inflammatory lesions usually show dye leakage on FFA, making it hard to distinguish neovascular from non-neovascular conditions. In cases of uveitis affecting the retina, the RPE, and the choroid, the absence of leakage in OCT-A allows for better visualization of the vascular network, therefore distinguishing a neovascular process from an inflammatory one [9]. In APMPPE, OCT-A shows areas of flow deficit at the level of the choriocapillaris, which correlate closely with areas of outer retinal disruption on OCT. These same areas would manifest as early hypofluorescent on FFA and throughout all phases of ICGA [10]. Furthermore, the areas of outer retinal changes on OCT correspond to larger areas of choriocapillaris flow deficit on OCT-A. This finding would be in keeping with the hypothesis that the sequence of causative events in APMPPE starts with ischemia at the level of the choriocapillaris, which in turn affects the outer retina and RPE with photoreceptor disruption [11]. On follow-up, OCT-A demonstrates a reversible inner choroidal hypoperfusion, indicating normalization of the choroidal vasculature [2].

Another non-invasive imaging modality is FAF, which provides a measure of RPE function. In APMPPE, patterns and characteristics of autofluorescence have been recognized as an indicator of disease activity [12]. Placoid lesions show initial hypoautofluorescence, which corresponds to the hyper-reflectance of the outer retinal layers on spectral-domain OCT (SDOCT), possibly due to a masking effect. This is followed by a gradual increase in hyperautofluorescence due to disruption of the RPE layer and a reduction of the overlying infiltrate. Later stages in the disease course are characterized by a mixed pattern of hypoautofluorescence and hyperautofluorescence and might be followed by a more homogenous hypoautofluorescent pattern due to photoreceptor layer and RPE atrophy [13].

In conclusion, multimodal imaging can subdivide disease progression in APMPPE into four distinct consecutive phases as proposed by Burke et al. in 2017 [14]: 

1. Choroidal phase: choriocapillaris hypoperfusion detected with OCT-A and confirmed with ICGA. In this phase, SDOCT and FAF are normal.

2. Chorioretinal phase: development of active lesions on FFA, which show early hypofluorescence and late hyperfluorescence, as well as persistent hypofluorescence of lesions across all ICGA phases and choriocapillaris hypoperfusion on OCT-A. OCT shows disruption of the outer retinal layers and ellipsoid zone, with hyperreflective material at the level of the OPL/ONL and RPE layers. The lesions are predominantly hypoautofluorescent on FAF.

3. Transitional phase: OCT confirms thinning and disruption of outer retinal layers with hypoperfusion of the choriocapillaris on OCT-A, and progressive hyperautofluorescence on FAF.

4. Resolution phase: Partial restoration of the outer retina with focal areas of photoreceptors/RPE atrophy visible on OCT and predominantly hypoautofluorescent changes visible on FAF with a variable restoration of choriocapillaris flow on OCT-A.

This report is limited by its nature as a two-case series, which restricts the strength of evidence supporting the use of non-invasive imaging in APMPPE diagnosis and follow-up. The absence of serial dye-based angiography further limits direct comparison with established gold-standard techniques. Nevertheless, the consistent multimodal imaging patterns observed here highlight the potential value of OCT, OCT-A, and FAF in monitoring APMPPE. These observations should be interpreted as exploratory, and they highlight the need for larger studies to validate these findings and define the role of non-invasive imaging in routine clinical practice.

## Conclusions

Non-invasive multimodal imaging, including OCT, FAF, and OCT-A, offers a reliable and comprehensive means of monitoring disease activity, progression, and treatment response in APMPPE. Characteristic changes across these imaging modalities correlate closely with clinical improvement, providing valuable biomarkers of disease resolution. The ability to track structural and vascular changes without the risks or inconvenience of dye-based angiography supports the growing role of these techniques in both acute management and long-term follow-up of APMPPE.

Looking forward, the identification of reproducible OCT-A and FAF biomarkers may help standardize disease-activity scoring not only for APMPPE but potentially for other inflammatory choriocapillaropathies.
